# Novel Peptides LFLLP and DFFL from Jack Bean Protein Hydrolysates Suppress the Inflammatory Response in Lipopolysaccharide-Stimulated RAW 264.7 Cells

**DOI:** 10.3390/foods13193198

**Published:** 2024-10-08

**Authors:** Bambang Dwi Wijatniko, Yoshiki Ishii, Makoto Hirayama, Takuya Suzuki

**Affiliations:** 1 Graduate School of Integrated Sciences for Life, Hiroshima University, Higashihiroshima 739-8528, Japan; bambangdw92@ugm.ac.id (B.D.W.); m231345@hiroshima-u.ac.jp (Y.I.); hirayama@hiroshima-u.ac.jp (M.H.); 2 Department of Food and Agricultural Product Technology, Universitas Gadjah Mada, Yogyakarta 55281, Indonesia

**Keywords:** lipopolysaccharide, macrophage cells, jack bean protein hydrolysates, anti-inflammatory peptides, tumor necrosis factor-α, Alcalase

## Abstract

The production of inflammatory cytokines such as tumor necrosis factor (TNF)-α by activated macrophage cells plays an important role in the development of intestinal inflammation. The present study investigated the anti-inflammatory effect of the protein hydrolysates prepared from the jack bean (JBPHs), *Canavalia ensiformis* (L.) DC, using the enzyme Alcalase, in a murine macrophage model, RAW 264.7 cells, which were stimulated by lipopolysaccharides. JBPHs reduced the TNF-α expression at the protein and mRNA levels through the downregulation of cellular signaling pathways involved in nuclear factor kappa B (NF-κB), extracellular signal-regulated kinase (ERK), and p38. A combination of mass spectrometry and in silico approaches identified 10 potential anti-inflammatory peptides in the JBPHs, including LFLLP and DFFL. Interestingly, while LFLLP targeted the NF-κB pathway, DFFL targeted p38 and ERK to suppress the TNF-α production in the RAW 264.7 cells. In addition, LFLLP and DFFL were localized in the cytosol of the cells. These results demonstrated that LFLLP and DFFL were incorporated by RAW 264.7 cells and, at least in part, contributed to the reduction in TNF-α by JBPHs. These peptides isolated from JBPHs may well be utilized as new alternatives to alleviate intestinal inflammation.

## 1. Introduction

Intestinal inflammation, commonly referred to as inflammatory bowel disease (IBD), encompasses chronic conditions such as Crohn’s disease and ulcerative colitis, both of which have a significant impact on patients’ quality of life. These disorders are distinguished by recurrent inflammation of the gastrointestinal tract, which frequently manifests as severe abdominal pain, diarrhea, weight loss, and fatigue. The global incidence and prevalence of IBD have been observed to increase steadily, particularly in industrialized nations. However, cases are also rising in developing countries as they undergo rapid urbanization and lifestyle changes. The impact of intestinal inflammation extends beyond the medical sphere, affecting social and economic aspects as well [1]. These diseases often affect young individuals, leading to long-term healthcare needs, a loss of productivity, and a strain on healthcare systems. Despite advances in medical therapies, a cure for IBD remains elusive, underscoring the critical need for research to elucidate its underlying mechanisms and develop more effective treatments [2].

A key mediator of this inflammatory response is the tumor necrosis factor (TNF)-α, a pro-inflammatory cytokine predominantly produced by activated macrophages in the intestinal mucosa [3]. Macrophages, as central players in the innate immune response, become activated in the presence of lipopolysaccharide (LPS), a component of the outer membrane of Gram-negative bacteria. Upon LPS binding to the Toll-like receptor 4 (TLR4) on macrophages, a cascade of intracellular signaling events is triggered, including the activation of NF-κB and MAPKs pathways, which promote the expression of pro-inflammatory genes [4]. This signaling ultimately leads to the secretion of TNF-α and other cytokines that exacerbate intestinal inflammation, contributing to the pathogenesis of conditions like IBD [5]. 

In recent years, the use of nutraceuticals derived from natural compounds has emerged as a new approach in the management of IBD due to their promising safety and fewer side effects during long-term interventions. A growing body of evidence suggests that peptides derived from protein hydrolysates often exert different biological functions, such as anti-oxidative, anti-hypertensive, and anti-inflammatory activities [6]. It seems that the biological activities of peptides liberated through the enzymatic digestion of proteins are influenced by their molecular weight, hydrophobicity, and net charge [7]. Previous studies have shown that some food-derived protein hydrolysates and peptides such as mung bean and walnut protein hydrolysates mitigate intestinal inflammation by downregulating the production of pro-inflammatory cytokines in lipopolysaccharide (LPS)-stimulated RAW 264.7 macrophage cells [8,9]. Although the precise mechanisms underlying the peptide-mediated anti-inflammatory effects are still unclear, these peptides are possibly delivered to the intestinal macrophages and interact with target biomolecules, such as membrane receptors and intracellular signaling proteins [10]. 

The jack bean (JB, *Canavalia ensiformis* (L.) DC) is a legume commonly cultivated in Java, Borneo, and the Sulawesi islands of Indonesia. The JB is characterized by its long, oval shape and white peel and its seed is yellowish with a double cotyledon. The JB is highly productive and rich in protein. Despite its considerable potential as a food ingredient, the JB is rarely utilized in this capacity due to the complexity of its processing. The thick pericarp of the beans must be peeled after soaking in water for 24 h, which is a significant obstacle to its incorporation into food products [11]. The strong beany flavor of the JB may contribute to a reduced acceptance of JB products with sensory attributes, such as tempeh. Furthermore, the presence of anti-nutritional substances in the JB, including hydrogen cyanide, trypsin inhibitor, phytic acid, and tannin, impede the absorption of nutrients [12]. Although previous studies have shown some techniques to reduce the levels of these anti-nutritional substances in the JB [12,13], the utilization of the JB remains low, indicating a discrepancy between its potential production and functionality.

The proteins of the JB are composed primarily of canavalin as its major protein storage, but it also contains significant amounts of concanavalin, a lectin that binds specifically to mono-, oligo-, and polysaccharides with terminal nonreducing α-D-mannopyranosyl, α-D-glucopyranosyl, or β-D-fructofuranosyl residues. The essential amino acid content in the JB is significantly greater than that found in common legumes (*Vigna mungo* (L.) Hepper, *Vigna radiata* (L.) R. Wilczek, *Cicer arietinum* L., and *Cajanus cajan* (L.) Millsp) [11]. Interestingly, the presence of hydrophobic, aromatic, and positively charged amino acids in JB proteins is relatively high [12]. We previously demonstrated that peptides derived from JB protein hydrolysates (JBPHs), which were prepared using pepsin–pancreatin enzymes, reduced the TNF-α-induced IL-8 production in human intestinal Caco-2BBe cells via suppression of the p65/p38/JNK signaling pathway [14]. Considering that the digestive specificity of enzymes influences the production of bioactive peptides, we conceived that additional bioactive peptides could be produced from the JB using other enzymes such as Alcalase. The present study aimed to investigate the anti-inflammatory effect of JBPHs-derived peptides and the underlying mechanisms in a well-validated macrophage model, RAW 264.7 macrophage cells. In addition, an in silico approach was used to identify the potential peptides responsible for the anti-inflammatory activity in RAW 264.7 cells.

## 2. Materials and Methods

### 2.1. Materials

Jack bean seeds were obtained from a local farmer in Wonogiri, Central Java, Indonesia. The seeds were ground and sieved through a 250 μm mesh sieve to obtain the JB powder. Two peptides, LFLLP and DFFL, were obtained from Eurofins Genomics (Tokyo, Japan). Alcalase^®^, lipopolysaccharide, and acetonitrile were obtained from Merck Millipore (Burlington, MA, USA). Fetal bovine serum (FBS) and trypsin–EDTA solution were obtained from Thermo Fisher Scientific (Waltham, MA, USA). Penicillin−streptomycin (10,000 U/mL) was obtained from Nacalai Tesque (Kyoto, Japan). Trifluoroacetic acid (TFA) was purchased from FUJIFILM Wako Pure Chemical (Osaka, Japan). An enzyme-linked immunosorbent assay (ELISA) kit to determine TNF-α was obtained from R&D Systems (Minneapolis, MN, USA). Eukitt^®^ quick-hardening mounting medium was purchased from Sigma Aldrich (St. Louis, MO, USA). All other chemicals were purchased from FUJIFILM Wako Pure Chemical and Nacalai Tesque.

### 2.2. Jack Bean Protein Extraction

The JB protein isolate was prepared according to a previous procedure [15]. Briefly, JB powder was defatted with petroleum ether with a ratio of 1:2 (*m*/*v*) by continuous stirring for 30 min, followed by removal of the solvent with filter paper. The fat extraction was carried out twice. For isolation of the JB protein, the defatted JB powder was dispersed in ultra-pure water (1:4, *w*/*v*), and the pH was adjusted to 8.0 with 1 mol/L NaOH. The mixture was vigorously stirred for 1 h and centrifuged at 10,000× *g* for 15 min at 4 °C. The supernatant was collected, and the pH was adjusted to 4.5 with 1 mol/L HCl to coagulate the protein. The JB protein was obtained by centrifugation at 5000× *g* for 15 min at 4 °C and stored at −30 °C. The protein concentration in the JB protein was measured using a Bicinchoninic Acid (BCA) protein assay kit (FUJIFILM Wako Pure Chemical).

### 2.3. Preparation of Jack Bean Protein Hydrolysates 

JB protein extracts were dissolved in ultra-pure water at the protein concentration of 5% (*w*/*v*). The pH of the mixture was adjusted to 8 with 1 mol/L NaOH followed by hydrolysis with Alcalase at an enzyme-to-substrate (E/S) ratio of 1:50 (60 °C) in a shaking (150 rpm) water bath at different hydrolysis times (30, 60, 120, and 240 min). To obtain the supernatant, the enzyme activity was stopped by heating in boiling water for 10 min, followed by centrifugation at 2380× *g* for 15 min. The protein concentration in the supernatant was determined using a BCA assay. To isolate the peptides and remove the salt in the supernatant, Sep-Pak C18 Plus Short Cartridge (Waters, Milford, MA, USA) was used with 2% acetonitrile + 0.1% TFA for conditioning and washing and 65% acetonitrile + 0.1% TFA for elution. The desalted eluate was freeze-dried (FDU-1200, EYELA, Tokyo, Japan) and designated as the JBPHs powder. The JBPHs powder was stored at −30 °C until further use.

### 2.4. Peptide Fractionation of Jack Bean Protein Hydrolysates

#### 2.4.1. Ultrafiltration of Jack Bean Protein Hydrolysates

Peptides in JBPHs were fractionated through ultrafiltration membranes with a molecular weight cut-off (MWCO) of 3 kDa (Amicon^®^ Ultra-15 3K, Merck Millipore). The JBPHs powder was dissolved in ultra-pure water and placed into an Amicon^®^ Ultra-15 3K device, followed by centrifugation at 2380× *g* for 30 min. The permeate (filtrated fraction) was freeze-dried and stored at −30 °C for further purification and analysis.

#### 2.4.2. Reversed-Phase High-Performance Liquid Chromatography

The filtrated fraction was purified by Reversed-Phase High-Performance Liquid Chromatography (RP-HPLC) (JASCO, Tokyo, Japan) using a TSKgel ODS-120T column (5 µm, 4.6 mm i.d. × 250 mm, TOSOH, Tokyo, Japan). The fraction was eluted by water (A) and 100% acetonitrile (B) with TFA 0.1% for 40 min at a flow rate of 1 mL/min. The gradient elution program was carried out based on the following steps: 0–100% B for 2–35 min and equilibrate with 100% B for 35–40 min. Eluted peptides were captured by a UV VIS detector at 220 nm and collected according to the retention time. Eight fractions were collected based on the retention time and then freeze-dried to determine their anti-inflammatory activity. 

### 2.5. Peptide Identification by Mass Spectrometry and in Silico Analysis

After RP HPLC separation, the peptide fractions were resuspended in water + 0.1% TFA and analyzed by nano-liquid chromatography mass spectrometry (LC-MS/MS) for peptide identification. The peptides were separated using an Ultimate 3000 RSLCnano system and an LTQ Orbitrap XL (Thermo Fisher Scientific). The peptides were concentrated with trap columns (PepMap^TM^ Neo 5 μm C18 300 μm × 5 mm, Thermo Fisher Scientific) and separated in line using a RP-C18 column (3 µm, 75 µm i.d. × 120 mm, Nikkyo Technos, Tokyo, Japan) at a flow rate of 200 nL/min under a gradient elution of 0.1% formic acid in water (A) and acetonitrile and 0.1% formic acid (B) with an elution program as follows: 0–3 min, 0–4% B; 3–30 min, 4–75% B; 30–31 min, 75–90% B; 31–35 min, 90% B; and 35–45 min, 4% B. The mass spectrometry data were acquired in a mass range of 100–1000 *m*/*z*. Peptide sequences were recognized based on de novo sequencing incorporated with PEAKS X software (version 10.0, Bioinformatics Solutions Inc., Waterloo, ON, Canada). From the identified peptides, only peptides with an average local confidence (ALC) ≥ 85% were selected for in silico analysis using the BIOPEP-UWM database of anti-inflammatory peptides (https://biochemia.uwm.edu.pl/biopep/start_biopep.php (accessed on 20 May 2024)), PreTP-EL (http://bliulab.net/PreTP-EL (accessed on 20 May 2024)), and PeptideRanker (http://distilldeep.ucd.ie/PeptideRanker/ (accessed on 20 May 2024)). 

### 2.6. Cell Culture

Cells of the mouse macrophage cell line RAW 264.7 (TIB-71, American Type Culture Collection, Manassas, VA, USA) were cultured in Dulbecco’s modified Eagle’s medium (DMEM, Nacalai Tesque) supplemented with 100 mL/L FBS and penicillin–streptomycin. The cells were maintained at 37 °C under 5% CO_2_. The experiments were carried out between passage numbers 4 and 25. 

### 2.7. Cell Viability Assay

RAW 264.7 cells were seeded in a 96-well plate (0.2 × 10^3^ cells/cm^2^), treated with and without JBPHs at various concentrations (250–1500 µg/mL) for 24 h. The viability of RAW 264.7 cells was analyzed using a Cell Counting Kit-8 (CCK-8, Dojindo, Kumamoto, Japan), according to the manufacturer’s instructions. The cell viability was shown as a percentage relative to the control group based on absorbance at 450 nm.

### 2.8. Evaluation of the Anti-Inflammatory Activity of Jack Bean Protein Hydrolysates in RAW 264.7 Cells

RAW 264.7 cells were seeded in a 48-well plate (0.3 × 10^4^ cells/cm^2^). After 3 days, the cells were treated with LPS (1 ng/mL) and incubated for 24 h. JBPHs (at hydrolysis times of 30, 60, 120, and 240 min, and concentrations of 250, 500, and 1500 μg/mL) was added into the cell culture media 6 h prior to the LPS administration. The TNF-α production in the cell culture media was determined by ELISA (Mouse TNF-α DuoSet ELISA, R&D systems), according to the manufacturer’s instructions. To elucidate the anti-inflammatory mechanisms of JBPHs, the cells were subjected to a quantitative reverse transcription polymerase chain reaction (qRT-PCR), immunoblot, and immunofluorescence analyses, as described below. Following procedures similar to those used when JBPHs was analyzed, the anti-inflammatory activities of different fractions from JBPHs and two peptides, LFLLP and DFFL, were also examined. 

### 2.9. Quantitative Reverse Transcription Polymerase Chain Reaction Analysis 

RAW 264.7 cells were seeded in a 24-well plate (0.6 × 10^4^ cells/cm^2^). After 3 days, the cells were treated with LPS (1 ng/mL) and incubated for 3 h. JBPHs 240 min (at concentrations of 500 and 1500 μg/mL) was added into the cell culture media 6 h prior to the LPS administration. Three hours after LPS administration, RAW 264.7 cells were washed with cold phosphate-buffered saline (PBS) and the total RNA was isolated using Sepasol^®^-RNA I Super G (Nacalai Tesque). cDNA was synthesized from RNA by reverse transcription using a ReverTra Ace qPCR RT kit (TOYOBO, Osaka, Japan), according to the manufacturer’s instructions. The RT-PCR reaction was conducted using Brilliant III Ultra-Fast SYBR Green qPCR Mix (Agilent, Santa Clara, CA, USA) in a StepOne Real-Time PCR system (Thermo Fisher Scientific). The relative mRNA expression of *Tnfa* was calculated using the 2^−ΔΔCt^ method, and the target gene expression was normalized to ribosomal protein S28 (*Rps28*) as the reference gene. The primer sequences of *Tnfa* and *Rps28* used are listed in Table S1.

### 2.10. Immunoblot Analysis

RAW 264.7 cells were seeded in a 24-well plate (0.5 × 10^4^ cells/cm^2^). After 3 days, the cells were treated with LPS (1 ng/mL) and incubated for 30 min. JBPHs 240 min (at concentrations of 500 and 1500 μg/mL) was added into the cell culture media 6 h prior to the LPS administration. An amount of 30 min after LPS administration, RAW 264.7 cells were washed with cold PBS and lysed in a lysis buffer, which contained sodium dodecyl sulfate (10 g/L), Triton X-100 (10 mL/L), and sodium deoxycholate (1 g/L) in 30 mmol/L Tris with protease and phosphatase inhibitors at pH 7.4. The protein concentration was measured using a BCA assay. Samples were mixed with half a volume of Laemmli sample buffer (3× concentrated) and heated to 95 °C for 10 min [16]. Subsequently, 20 µg of protein was separated by sodium dodecyl sulphate polyacrylamide gel electrophoresis at the appropriate voltage, and the protein band was transferred to polyvinylidene difluoride membranes (Immobilon^®^-P, Merck Millipore). The membranes were blocked with 50 mg/mL of skim milk for 1 h and incubated with primary antibodies at 4 °C for 16 h. After washing five times with TBST buffer, the membranes were incubated for 1 h with a horseradish peroxidase-conjugated anti-rabbit IgG secondary antibody at a dilution of 1:15,000. Specific bands were visualized using an enhanced chemiluminescence detection reagent (Western-Lightning Plus-ECL, PerkinElmer, Waltham, MA, USA) in an Amersham Imager 680 apparatus (Cytiva, Uppsala, Sweden). The specific band density was calculated by ImageJ (National Institutes of Health, Bethesda, MD, USA). Protein expression was normalized to that of the reference using Ponceau S staining [17]. The information of the antibodies is shown in Table S2.

### 2.11. Immunofluorescence Microscopy

The phospho-p38 protein expression in RAW 264.7 cells was examined using immunofluorescence microscopy. RAW 264.7 cells were seeded in a 6-well plate (0.5 × 10^4^ cells/cm^2^). After 3 days, the cells were treated with LPS (1 ng/mL) and incubated for 30 min. The cells were incubated with or without 500 µg/mL of JBPHs 240 min for 6 h before LPS administration. An amount of 30 min after LPS administration, the cells were washed with cold PBS and fixed with 4% paraformaldehyde (PFA) for 10 min. Subsequently, the cells were permeabilized with 0.2% Triton X-100 for 5 min, blocked with 5% bovine serum albumin, and incubated with an anti-phospho-p38 protein antibody at 4 °C for 16 h. Next, the cells were washed three times with PBS and then incubated with Alexa Fluor 488-conjugated anti-rabbit IgG and DAPI for 1 h. The specimens were preserved in the mounting fluid and the immunofluorescence signals were visualized using a Leica DMI6000 B fluorescence microscope (Leica, Wetzlar, Germany).

### 2.12. Visualization of LFLLP and DFFL Peptides in RAW 264.7 Cells

To visualize the cellular localization of peptides, LFLLP and DFFL were conjugated with fluorescein isothiocyanate isomer-I (FITC-I, Dojindo). Briefly, the LFLLP and DFFL were mixed with FITC-I in a carbonate buffer at pH 9.5 for 2 h. To purify the FITC-conjugated peptides, the mixture was applied to a Sep-Pak C18 cartridge. RAW 264.7 cells were seeded in a 6-well plate (0.5 × 10^4^ cells/cm^2^). After 3 days, the cells were incubated with FITC-conjugated peptides (LFLLP and DFFL, 200 µmol/L) for 24 h. The cells were then washed with cold PBS and fixed with 4% PFA for 10 min. Lastly, the cells were washed three times with PBS and mounted on microscope slides using a mounting fluid containing DAPI, and the fluorescence signals were visualized using a Leica DMI6000 B fluorescence microscope (Leica).

### 2.13. Statistical Analysis

Statistical analyses were carried out using JMP software (version 16; SAS Institute Inc., Cary, NC, USA). Data are presented as the means ± the SEM. One-way analysis of variance (ANOVA) was used to determine the statistical significance, followed by the Dunnett or the Tukey–Kramer post hoc test. *p* values < 0.05 were considered statistically significant.

## 3. Results

### 3.1. Effect of Jack Bean Protein Hydrolysates on the Viability of RAW 264.7 Cells

**Figure 1:**
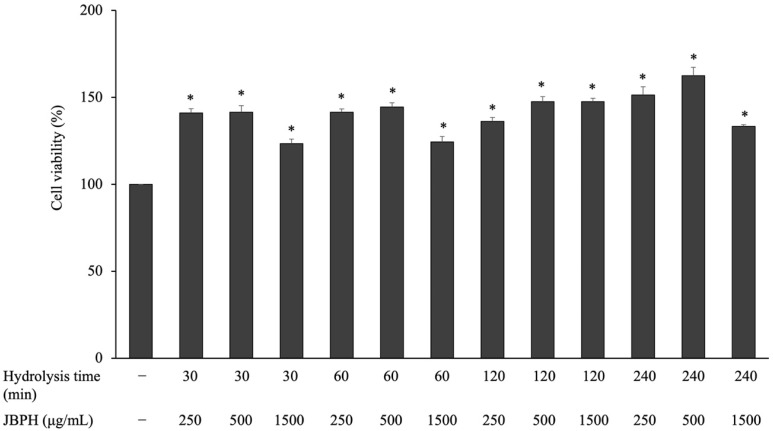
Effect of jack bean protein hydrolysates on the viability of RAW 264.7 cells. RAW 264.7 cells were incubated with JBPHs for 24 h and the cell viability was evaluated using a commercially available kit. Values are presented as the means ± the SEM (n = 6). The asterisks indicate *p* < 0.05 compared with the control, as determined using the Dunnett post hoc test.

Dietary peptides should not show cytotoxicity to macrophage cells. Treatment of cells with JBPHs at concentrations in the range of 250–1500 µg/mL for 24 h did not reduce the viability of RAW 264.7 cells (Figure 1).

### 3.2. Anti-Inflammatory Effect of Jack Bean Protein Hydrolysates

**Figure 2:**
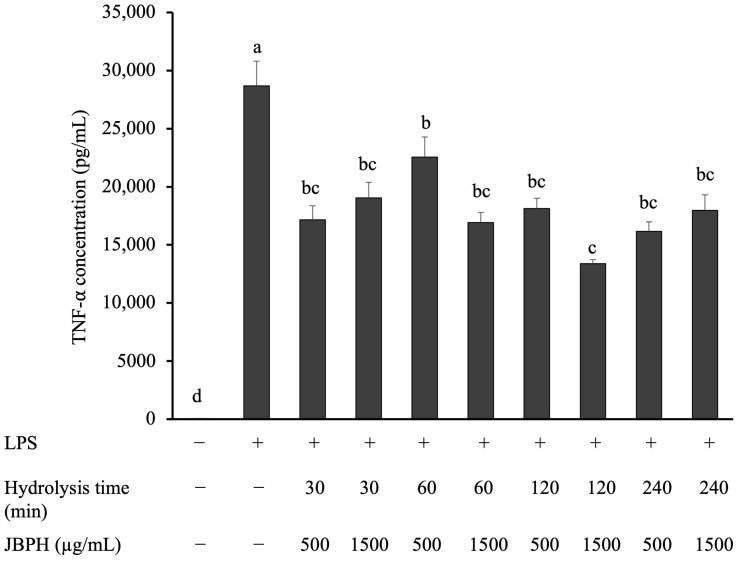
Effect of jack bean protein hydrolysates on TNF-α protein levels in RAW 264.7 cells. RAW 264.7 cells were incubated with and without JBPHs in the presence and absence of 1 ng/mL of LPS for 24 h. The TNF-α protein production was determined by ELISA. Values are shown as the means ± the SEM (n = 6). Means not marked by a common letter are significantly different, as determined using the Tukey–Kramer post hoc test (*p* < 0.05).

TNF-α produced from activated macrophages has a pivotal role in intestinal inflammation [10]. Figure 2 indicates that RAW 264.7 cells treated with 1 ng/mL LPS showed a notable increase in TNF-α production when compared with control cells, exhibiting the activation of the inflammatory response. Pretreatment with JBPHs at all hydrolysis times reduced the production of TNF-α in a roughly similar manner. Since the hydrolysis time is often associated with the degree of hydrolysis [18,19], small peptides may contribute to the JBPHs-mediated reduction in TNF-α. Consequently, JBPHs prepared by hydrolysis for 240 min (JBPHs 240 min) was selected for further experiments.

**Figure 3:**
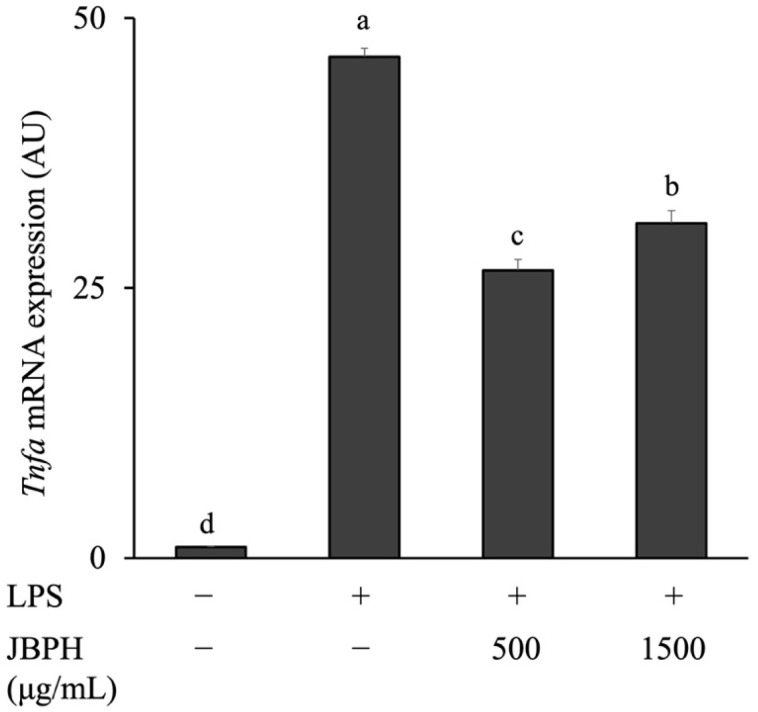
Effect of jack bean protein hydrolysates on *Tnfa* mRNA levels in RAW 264.7 cells. RAW 264.7 cells were incubated with and without JBPHs 240 min in the presence and absence of 1 ng/mL of LPS for 3 h. The *Tnfa* mRNA expression was determined by qRT-PCR. Values are presented as the means ± the SEM (n = 6). Means not marked by a common letter are significantly different, as determined using the Tukey–Kramer post hoc test (*p* < 0.05).

To examine whether the reduction in TNF-α by JBPHs was transcriptionally regulated, the cells incubated with 500 and 1500 μg/mL JBPHs 240 min were subjected to qRT-PCR analysis. Consistent with the TNF-α protein production, the LPS administration upregulated the *Tnfa* mRNA expression and JBPHs reduced the *Tnfa* mRNA expression with a pronounced effect at 500 μg/mL (Figure 3).

### 3.3. Cellular Mechanism of the Anti-Inflammatory Effect of Jack Bean Protein Hydrolysates

**Figure 4:**
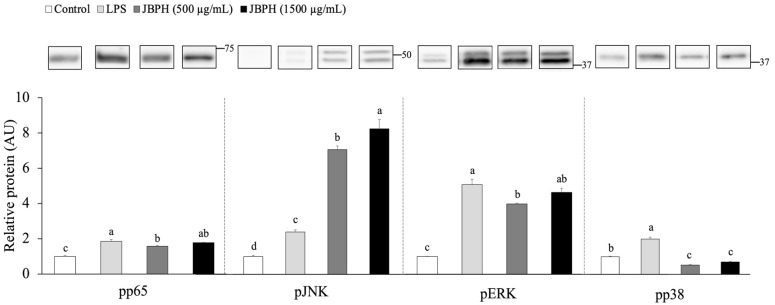
Effect of jack bean protein hydrolysates on phosphorylation of signaling molecules. RAW 264.7 cells were incubated with and without JBPHs 240 min in the presence and absence of 1 ng/mL of LPS for 30 min. Phosphorylation of p65, JNK, ERK, and p38 was determined by immunoblot analysis. Values are shown as the means ± the SEM (n = 6). Means not marked by a common letter are significantly different, as determined using the Tukey–Kramer post hoc test (*p* < 0.05).

**Figure 5:**
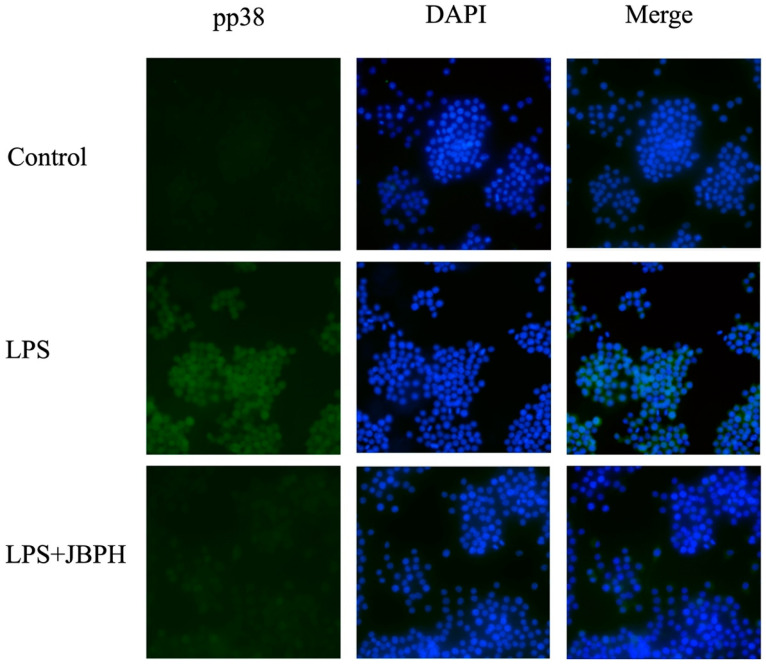
Effects of jack bean protein hydrolysates on the expression and localization of protein pp38. RAW 264.7 cells were incubated with and without 500 μg/mL of JBPHs 240 min in the presence and absence of 1 ng/mL of LPS for 30 min. Localization and expression of protein pp38 was determined by immunofluorescence microscopy.

Previous studies show that the LPS/TLR4 pathway activates several cellular signaling pathways such as NF-κB and MAPKs to upregulate the TNF-α expression in the macrophages [9,10]. An immunoblot analysis demonstrated that stimulation of RAW 264.7 cells with LPS for 30 min upregulated the phosphorylation of proteins p65, JNK, ERK, and p38, indicating the activation of inflammatory signaling pathways. The pretreatment of RAW 264.7 cells with JBPHs 240 min reduced the LPS-induced phosphorylation of proteins p65, ERK, and p38, but not JNK, with a noticeable effect at 500 μg/mL (Figure 4 and Figure S1). Furthermore, immunofluorescence microscopy showed that the phosphorylated protein p38 induced by LPS colocalized to the nucleus of RAW 264.7 cells, while it was downregulated by JBPHs 240 min (Figure 5). Taken together, the suppression of the p65/ERK/p38 signaling pathway was possibly associated with the JBPHs-mediated reduction in TNF-α transcription in RAW 264.7 cells. 

### 3.4. Anti-Inflammatory Effect of Peptides Fractionated from Jack Bean Protein Hydrolysates

**Figure 6:**
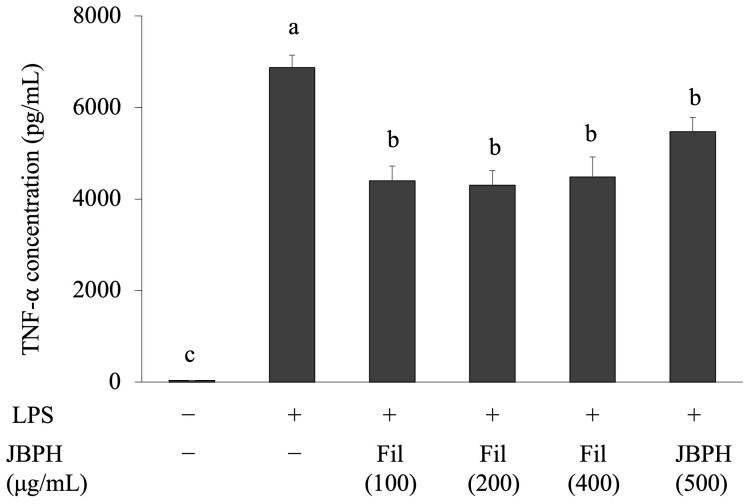
Effect of jack bean protein hydrolysates on the TNF-α protein levels in RAW 264.7 cells. RAW 264.7 cells were incubated with and without JBPHs 240 min and its filtrate fraction in the presence and absence of 1 ng/mL of LPS for 24 h. The filtrate fraction was prepared by ultrafiltration with 3 kDa MWCO. The TNF-α protein production in the filtrate fraction at different concentrations was determined by ELISA. Values are presented as the means ± the SEM (n = 5). Means not marked by a common letter are significantly different, as determined using the Tukey–Kramer post hoc test (*p* < 0.05).

The peptides in the JBPHs were separated by ultrafiltration using a 3 kDa MWCO filter. Based on the BCA protein assay, 15 and 85% of the peptides of JBPHs 240 min were distributed in the filtrate (<3 kDa) and retentate (>3 kDa) fractions, respectively. As mentioned earlier, we speculated that the small peptides contributed to the JBPHs-mediated effect. Therefore, the anti-inflammatory effect of the filtrate fraction was evaluated at different concentrations. Similar to the previous experiment, the original JBPHs reduced the LPS-induced TNF-α production in RAW 264.7 cells (Figure 6). The filtrate fraction, which included peptides < 3 kDa, also suppressed the LPS-induced TNF-α production. The suppression, even at a low concentration (100 μg/mL), was comparable with that of the original sample. 

**Figure 7:**
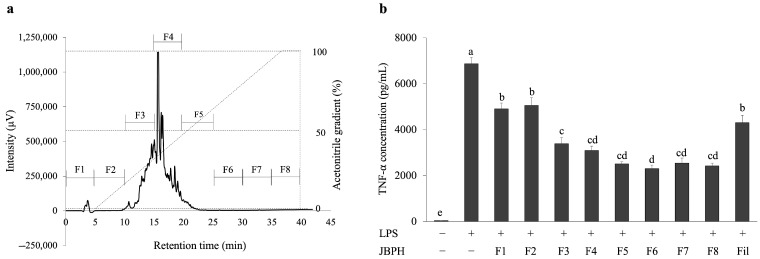
Separation of jack bean protein hydrolysates with Reversed-Phase High-Performance Liquid Chromatography and its effect on the TNF-α protein levels in RAW 264.7 cells. The chromatogram of JBPHs 240 min by RP-HPLC is shown. The dashed line indicates the acetonitrile gradient for elution (**a**). RAW 264.7 cells were incubated with and without JBPHs 240 min RP-HPLC fractions in the presence and absence of 1 ng/mL of LPS for 24 h. The TNF-α protein production was determined by ELISA (**b**). Values are presented as the means ± the SEM (n = 5). Means not marked by a common letter are significantly different, as determined using the Tukey–Kramer post hoc test (*p* < 0.05).

Next, the filtrate fraction of JBPHs was separated by RP-HPLC and eight fractions (F1–F8) were collected based on the retention time, as shown in Figure 7a. Similar to the previous experiment, the original filtrate fraction reduced the LPS-induced TNF-α production in RAW 264.7 cells (Figure 7b). Interestingly, all fractions obtained from the filtrate suppressed the LPS-induced TNF-α production in the cells, and notably fractions F6 and F8 showed the most potent suppression (Figure 7b). 

### 3.5. Identification of Potential Anti-Inflammatory Peptides

To identify the peptide sequence, fractions F6 and F8 obtained by RP-HPLC were subjected to de novo peptide sequencing with mass spectrometry. As a result, 21 and 7 peptides were identified in the F6 and F8 fractions, respectively. Based on an average local confidence (ALC) cut-off of ≥85% for de novo peptide sequencing quality, eight and two peptides were extracted from F6 and F8, respectively (Table 1), and subjected to the in silico approach. In the in silico approach, although no peptides were included in the BIOPEP-UWM database of anti-inflammatory peptides, four peptides, namely DFFL, LFVF, LFLLP, and VFPLL, marked scores higher than 0.8 in the PeptideRanker, indicating that these peptides were potentially bioactive. In addition, PreTP-EL, a computational tool designed to predict therapeutic peptides [20], predicted LFLLP as an anti-inflammatory peptide. According to the National Center for Biotechnology Information (NCBI) database, only one peptide (RSDPLYSN) in the F8 fraction was associated with the protein canavalin, but the parent proteins of the other nine peptides were not found.

### 3.6. Anti-Inflammatory Effect of Novel Peptides LFLLP and DFFL from Jack Bean Protein Hydrolysates 

**Figure 8:**
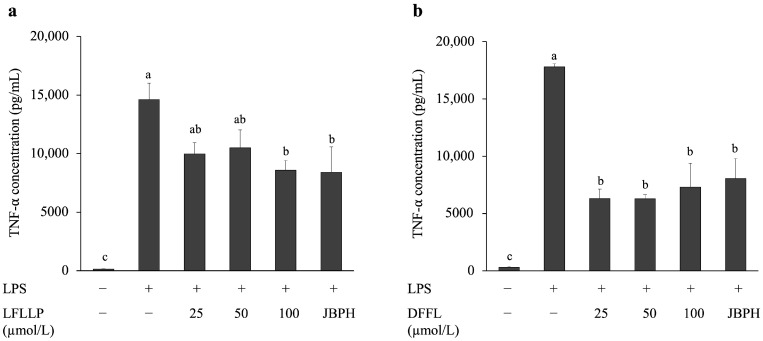
Effect of novel peptides LFLLP and DFFL on the TNF-α protein levels in RAW 264.7 cells. RAW 264.7 cells were incubated with and without the novel peptides LFLLP and DFFL in the presence and absence of 1 ng/mL of LPS for 24 h. The TNF-α protein production when novel peptides LFLLP (**a**) and DFFL (**b**) were present was determined by ELISA. Values are presented as the means ± the SEM (n = 5). Means not marked by a common letter are significantly different, as determined using the Tukey–Kramer post hoc test (*p* < 0.05).

Among the 10 peptides shown in Table 1, we focused on peptides LFLLP and DFFL from fraction F6, because DFFL marked the highest score in the PeptideRanker and LFLLP was the only one suggested as an anti-inflammatory peptide by PreTP-EL. LFLLP and DFFL were synthesized and LPS-stimulated RAW 264.7 cells were treated with them. Similar to the previous experiments, the original JBPHs reduced LPS-mediated TNF-α production in RAW 264.7 cells (Figure 8). In addition, the peptide LFLLP at 100 μmol/L reduced LPS-mediated TNF-α production. Peptide DFFL at concentrations ranging from 25 to 100 μmol/L also suppressed TNF-α production in RAW 264.7 cells.

### 3.7. Cellular Mechanism of the Anti-Inflammatory Effect of Novel Peptides LFLLP and DFFL from Jack Bean Protein Hydrolysates 

**Figure 9:**
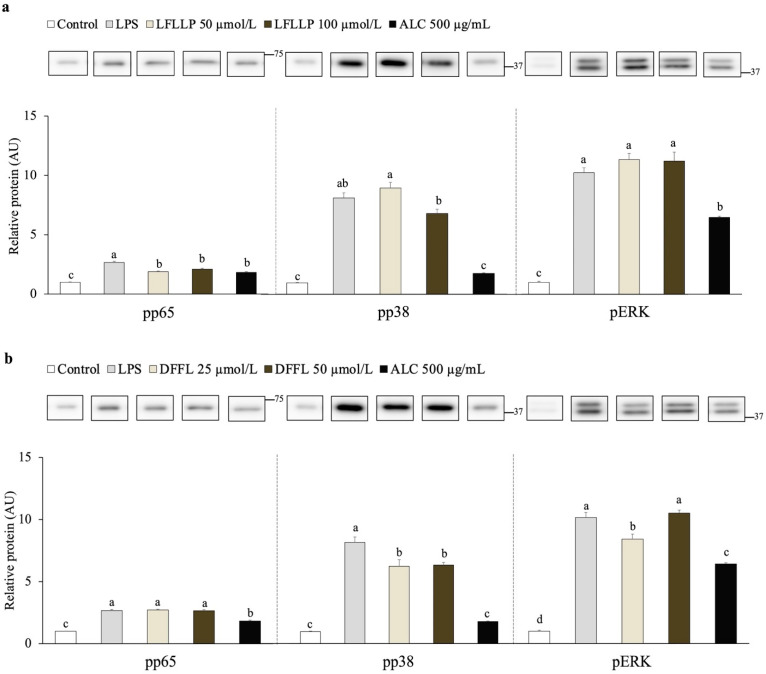
Effect of novel peptides LFLLP and DFFL on the phosphorylation of NF-κB and p38 signaling molecules. RAW 264.7 cells were incubated with and without novel peptides LFLLP and DFFL in the presence and absence of 1 ng/mL of LPS for 30 min. Phosphorylation of proteins p65, p38, and ERK after treatment with novel peptides LFLLP (**a**) and DFFL (**b**) were determined by an immunoblot analysis. Values are presented as the means ± the SEM (n = 6). Means not marked by a common letter are significantly different, as determined using the Tukey–Kramer post hoc test (*p* < 0.05).

Earlier experiments demonstrated that JBPHs suppressed the LPS-induced phosphorylation of NF-κB, p38, and ERK signaling molecules. An immunoblot analysis showed that the novel peptide LFLLP reduced the LPS-induced phosphorylation of protein p65, but not p38 and ERK, in RAW 264.7 cells (Figure 9a and Figure S2). In contrast to LFLLP, the peptide DFFL reduced the phosphorylation of proteins p38 and ERK, but not p65 (Figure 9b and Figure S2). These results indicated that LFLLP and DFFL targeted different signaling molecules to suppress the LPS-induced TNF-α production in RAW 264.7 cells.

### 3.8. Cellular Visualization of Novel Peptides LFLLP and DFFL in RAW 264.7 Cells

**Figure 10:**
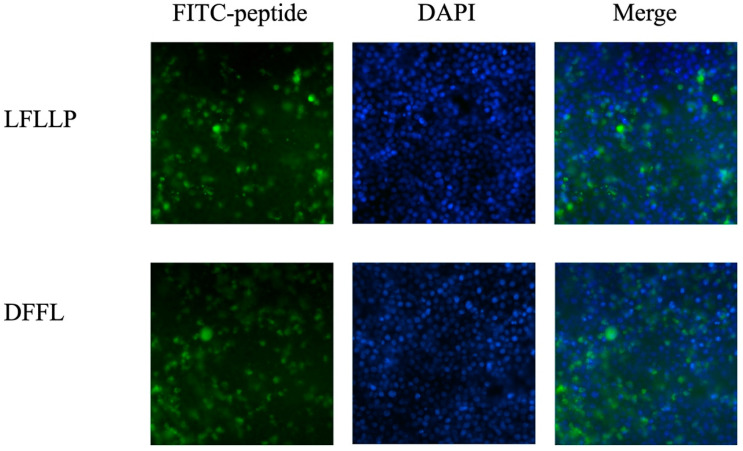
Cellular visualization of peptides LFLLP and DFFL. RAW 264.7 cells were incubated with 200 μmol/L FITC-conjugated LFLLP and DFFL for 24 h. Cellular localization of FITC-conjugated LFLLP and DFFL were visualized by fluorescence microscopy.

We hypothesized that the peptides LFLLP and DFFL were incorporated into the RAW 264.7 cells and suppressed the cellular inflammatory signaling. To investigate if LFLLP and DFFL were incorporated into RAW 264.7 cells, the cells incubated with FITC-conjugated peptides were observed with a fluorescence microscope. The fluorescence signal derived from FITC-conjugated LFLLP and DFFL were observed in most cells and the fluorescence intensity of the two peptides were roughly comparable, suggesting that the peptides were incorporated into the cells in a similar manner (Figure 10). The peptides were predominantly localized in the cytosols of RAW 264.7 cells, as indicated by the non-colocalization with DAPI. 

## 4. Discussion

The maintenance of the intestinal homeostasis is of great consequence to human health. The development of intestinal inflammation is attributed to the uncontrolled production of multiple proinflammatory cytokines, including TNF-α [21]. When LPS circulates extracellularly, the Toll-like receptor 4 (TLR4) interacts with LPS in a complex with myeloid differentiation factor 2 (MD-2), which is mainly expressed by immune cells. LPS is transferred to the TLR4–MD-2 complex at the cell surface by the accessory proteins lipopolysaccharide binding protein and CD14, which represents a crucial step in mediating LPS recognition by the immune system. Dimerization of the TLR4–MD-2 complex on the cell surface triggers activation of MYD88-dependent signaling and expression of transcriptional regulators such as NF-κB and activator protein-1 (AP-1), leading to the production of TNF-α [21]. The present study demonstrated that JBPHs prepared by Alcalase exhibited a reduction in TNF-α production in RAW 264.7 cells stimulated with LPS. The results indicated that the anti-inflammatory effect of JBPHs is associated with the regulation of certain cellular signaling pathways, including NF-κB and MAPKs. The anti-inflammatory mechanism of JBPHs mainly works through the inhibition of NF-κB and MAPKs. The present study suggested that JBPHs blocked the TNF-⍺-induced activation of NF-κB, including the inhibition of kinase enzymes of IKKα and MAPKK [22]. NF-κB is one of the essential transcriptional regulators that modulate the production of many cytokines and mediators, including TNF-α activation of mitogen-activated protein kinases (MAPKs) pathways, such as JNK, ERK, and p38. In addition, MAPKs as classical inflammatory signals are also mainly involved in the transcription of pro-inflammatory mediators [3,5]. Regulation of NF-κB and MAPKs might play a key role in the anti-inflammatory mechanism of JBPHs.

A combination of mass spectrometry and in silico approaches identified two novel anti-inflammatory peptides, LFLLP and DFFL. It was theorized that peptides, including LFLLP and DFFL, were incorporated into RAW 264.7 cells to interact with specific signaling molecules, thereby exerting anti-inflammatory activity. 

Intestinal inflammation is critically influenced by the overproduction of various inflammatory cytokines, with TNF-α playing a particularly pivotal role [9,10]. Elevated levels of TNF-α have been consistently observed in the lesions of patients with IBD and in corresponding animal models, underscoring its significance in the pathophysiology of these conditions [3]. The therapeutic efficacy of TNF-α-neutralizing antibodies in the treatment of ulcerative colitis, as demonstrated in both clinical and preclinical studies, further highlights its central role [23]. TNF-α, predominantly produced by activated macrophages, orchestrates the inflammatory response through multiple mechanisms. It induces apoptosis and the production of IL-8 and compromises the integrity of tight junctions in intestinal epithelial cells [24]. IL-8 recruits neutrophils, thereby exacerbating tissue damage through various inflammatory mediators. In addition, TNF-α enhances the inflammatory response by promoting the activity of Th1 and Th17 cells, leading to increased productions of IFN-γ and IL-17, and further driving the inflammatory cascade [25]. Therefore, the JBPHs-mediated reduction in TNF-α in macrophages could be a novel approach to maintain the intestinal homeostasis.

Two novel peptides, DFFL and LFLLP, were observed to suppress the phosphorylation of proteins p38/ERK and p65, respectively. This suggested that the JBPHs-mediated anti-inflammatory action may be attributed to the combined effect of these two peptides and potentially other peptides with a similar activity. Prior research has indicated that certain bioactive peptides interact with cell membrane receptors, including the calcium-sensing receptor and Toll-like receptors, to elicit their biological activity [10,26,27]. However, in the present work, fluorescence microscopy showed that DFFL and LFLLP were incorporated into RAW 264.7 cells and localized in the cytosol. While the present work did not elucidate the mechanisms underlying the incorporation of the peptides, it is known that macrophages have a high phagocytic ability, enabling them to engulf extracellular substances [28]. Thus, it is possible that DFFL and LFLLP exert their biological activity through the process of phagocytosis in RAW 264.7 cells.

The bioactivities of peptides are closely related to their structural characteristics, including molecular weight and amino acid composition [29,30]. Previous findings showed that peptides with low molecular weights (less than 1 kDa) tend to exhibit a higher anti-inflammatory activity [14,29]. Therefore, the present study focused on the peptide fraction below 3 kDa. Indeed, as evidenced in Table 1, the molecular weights of all the peptides were less than 1 kDa. Furthermore, these peptides contain a minimum of two hydrophobic amino acids, such as Ala (A), Val (V), Leu (L), Phe (F), Pro (P), and Tyr (Y). It has been demonstrated that the abundance of hydrophobic amino acids is associated with enhanced anti-inflammatory activities, as previously observed with a lupin peptide (GPETAFLR), two corn peptides (LLPSSQ and QLPY), and a millet bran peptide (VLLF) [31,32]. Although the precise role of the hydrophobic amino acid residues in the anti-inflammatory activity remains unclear, the presence of these residues seems to be involved in the inhibition of the inflammatory cascade in the cells [33].

Among the ten peptides shown in Table 1, the present work verified the anti-inflammatory activities of two peptides, LFLLP and DFLL, which were found in fraction 6. Nevertheless, it is conceivable that the remaining eight peptides, at least in part, also contribute to the JBPHs-mediated anti-inflammatory activity. In particular, only two peptides, NYGKLY and RSDPLYSN, have been identified by mass spectrometry. Although these two peptides did not mark high scores in the in silico program Peptideranker, they might be attributed to the anti-inflammatory activity of fraction 8, which was found in the RAW 264.7 cells. However, further studies are required to examine the anti-inflammatory activities of these remaining eight peptides. Moreover, the present study solely assessed the anti-inflammatory impact of JBPHs and its derived peptides in vitro, utilizing RAW 264.7 cells. Therefore, in future studies, it would be beneficial to validate these results with an appropriate murine model of intestinal inflammation.

## 5. Conclusions

JB protein hydrolyzed by Alcalase for 240 min liberated anti-inflammatory peptides. The peptides inhibited the TNF-α production by suppressing the NF-κB, ERK, and p38 signaling pathways in LPS-stimulated RAW 264.7 cells. In addition, an in silico approach identified two novel anti-inflammatory peptides, namely LFLLP and DFFL, in JBPHs. Our findings suggested that further exploration of plant-derived bioactive peptides derived from underutilized legume protein hydrolysates may be beneficial in promoting intestinal health. Hence, further studies are needed to elucidate the biological functions of these peptides in in vivo studies.

## Figures and Tables

**Table 1:** Potential anti-inflammatory peptides from fractions F6 and F8 by the in silico approach.

Fraction	Parent Protein	Peptide	*m*/*z*	RT	Area	ALC (%)	Error (ppm)	PeptideRanker Score ^a^	PreTP-EL Prediction ^b^
F6	Not found	DFFL	541.2656	21.19	6.93 × 10^6^	99	0	0.9809	Not predicted
	Not found	LFVF	525.3074	20.9	2.36 × 10^6^	99	0.6	0.9315	Not predicted
	Not found	LFLLP	602.3915	21.56	6.49 × 10^5^	94	0.5	0.8529	Anti-inflammatory peptide
	Not found	VFPLL	588.3756	20.7	5.48 × 10^5^	96	0.2	0.8413	Not predicted
	Not found	TFLL	493.3021	20.13	2.38 × 10^5^	92	0	0.7195	Not predicted
	Not found	VLLF	491.323	20.32	5.44 × 10^5^	97	0.5	0.6932	Not predicted
	Not found	FVPH	250.1369	13.88	9.48 × 10^4^	89	0.6	0.6694	Not predicted
	Not found	LALVL	528.3756	19.95	1.53 × 10^6^	87	0.1	0.2317	Not predicted
F8	Not found	NYGKLY	379.1975	15.88	1.21 × 10^4^	99	−0.1	0.4612	Not predicted
	Canavalin (SRDPIYSN)	RSDPLYSN	476.2299	14.54	6.34 × 10^3^	91	−0.5	0.3364	Not predicted

RT, retention time; ALC, average local confidence. ^a^ from PeptideRanker (http://distilldeep.ucd.ie/PeptideRanker/ (accessed on 20 May 2024)). ^b^ from PreTP-EL (http://bliulab.net/PreTP-EL (accessed on 20 May 2024)).

## Data Availability

The original contributions presented in the study are included in the article/Supplementary Materials, further inquiries can be directed to the corresponding author.

## Supplementary Materials

The following supporting information can be downloaded at https://www.mdpi.com/article/10.3390/foods13193198/s1: Figure S1: Representative of original western blotting (chemiluminescence) from JBPH; Figure S2: Representative of original western blotting (chemiluminescence) from peptides LFLLP and DFFL; Table S1: Primer sequences for qRT-PCR; Table S2: Information about antibodies used in this study.
