# Supplementary material for: Novel Peptides LFLLP and DFFL from Jack Bean Protein Hydrolysates Suppress the Inflammatory Response in Lipopolysaccharide-Stimulated RAW 264.7 Cells
Source: Foods. 2024 Oct 8;13(19):3198. doi: 10.3390/foods13193198 (PMC11482615; doi:10.3390/foods13193198)
Supplement: Supplementary file 1 [file foods-13-03198-s001.zip › foods-3216642-supplementary.pdf]

Supplementary materials

Novel Peptides LFLLP and DFFL from Jack Bean Protein Hydrolysates Suppress the Inflammatory Response in Lipopolysaccharide-stimulated RAW 264.7 Cells

Bambang Dwi Wijatniko[ab], Yoshiki Ishii[a], Makoto Hirayama[a], Takuya Suzuki[a*]

[a] Graduate School of Integrated Sciences for Life, Hiroshima University, Higashi-Hiroshima, 739-8528, Japan

[b]Department of Food and Agricultural Product Technology, Universitas Gadjah Mada, Yogyakarta, 55281, Indonesia

[*]**Correspondence to**:

Takuya Suzuki,

Graduate School of Integrated Sciences for Life, Hiroshima University,

Higashi-Hiroshima 739-8528, Japan.

E-mail : takuya@hiroshima-u.ac.jp

Tel : +81-82-424-7984

Fax : +81-82-424-7916

ORCiD: orcid.org/0000-0003-3709-543X

Table S1 Primer sequences for qRT-PCR

Target genes	Forward (5′ to 3′)	Reverse (5′ to 3′)
Mouse *Tnfa*	TCGTAGCAAACCACCAAGTG	CTTTGAGATCCATGCCGTTG
Mouse *Rps28*	ATCAAGCTGGCTAGGGTAACC	GGCCTTTGACATTTCGGATGA

Table S2. Information about antibodies used in this study

Antibody	Vender	Catalogue No.	Dilution	
			Immunoblot	Immunofluorescence
Rabbit anti-phospho SAPK/JNK (Thr 183/Tyr 182)	Cell Signaling Technology	9251S	1:8,000	NA
Rabbit-phospho-p38 (Thr180/Tyr182)	Cell Signaling Technology	4511S	1:3,000	1:800
Rabbit-phospho-Erk1/2 (Thr202/Tyr204)	Cell Signaling Technology	4370S	1:3,000	NA
Rabbit-phospho-p65 (Ser536)	Cell Signaling Technology	3033S	1:3,000	NA
Goat anti-rabbit IgG-HRP	SeraCare	074-1506	1:15,000	NA
Goat anti-rabbit IgG-AlexaFluor488	Abcam	Ab15007	NA	1:200

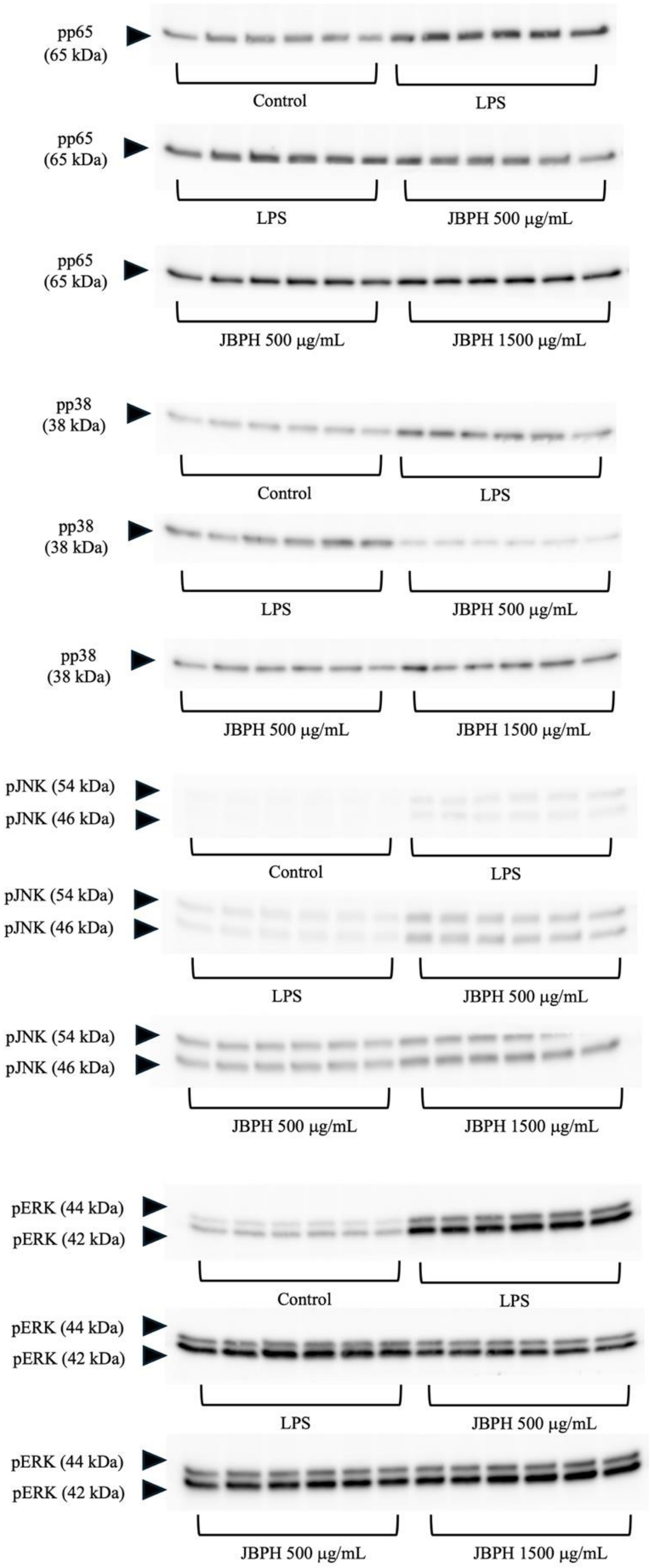

Figure **S1** Representative of original western blotting (chemiluminescence) from JBPH

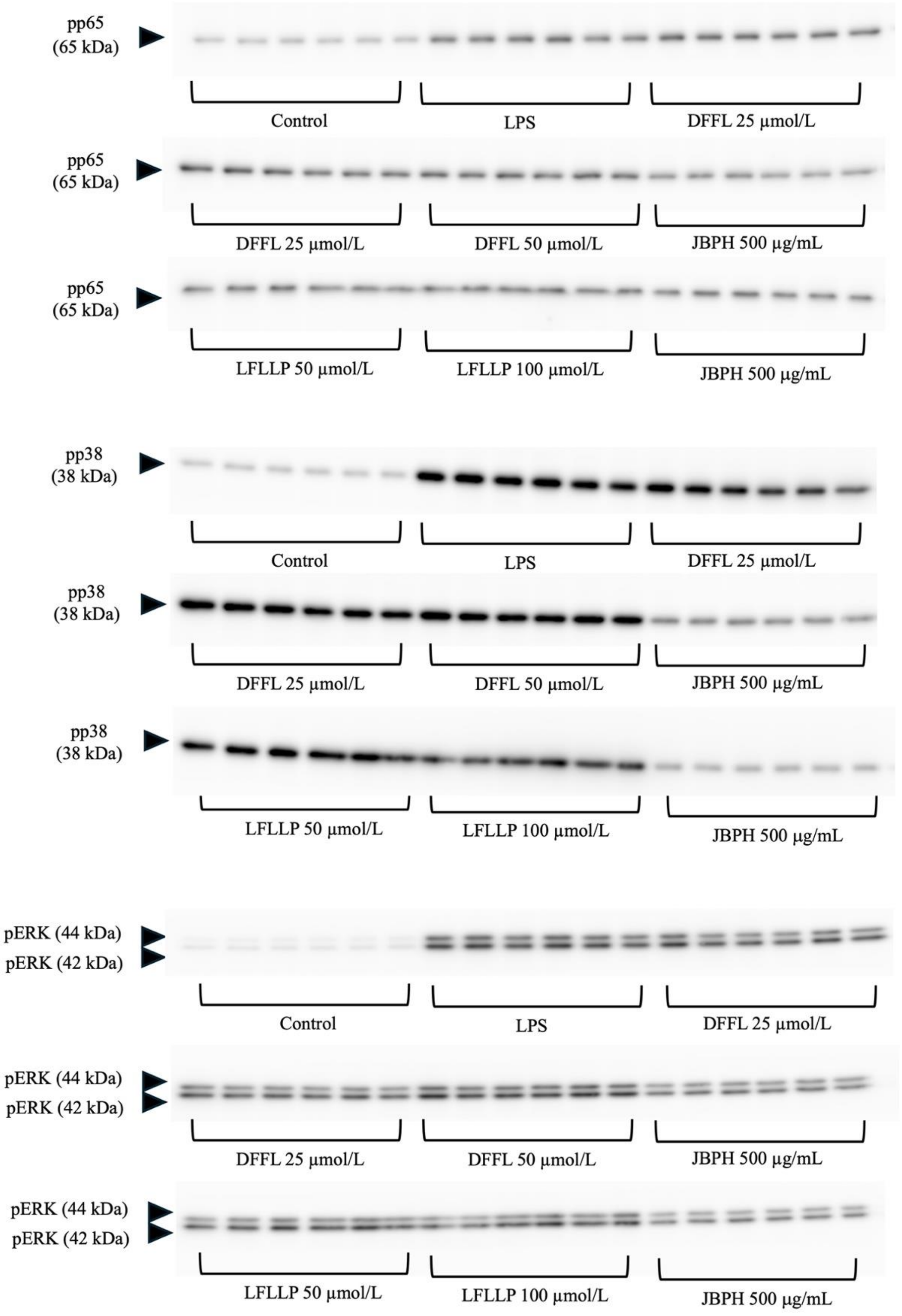

Figure **S2** Representative of original western blotting (chemiluminescence) from peptides LFLLP and DFFL
